# Impact of Diabetes on Complications, Long Term Mortality and Recurrence in 608,890 Hospitalised Patients with Stroke

**DOI:** 10.5334/gh.364

**Published:** 2020-02-06

**Authors:** Weronika A. Szlachetka, Tiberiu A. Pana, Somsak Tiamkao, Allan B. Clark, Kannikar Kongbunkiat, Kittisak Sawanyawisuth, Joao H. Bettencourt-Silva, Narongrit Kasemap, Mamas A. Mamas, Phyo K. Myint

**Affiliations:** 1 Ageing Clinical and Experimental Research (ACER) Team, Institute of Applied Health Sciences, School of Medicine, Medical Sciences and Nutrition, University of Aberdeen, Aberdeen, UK; 2 Neurology Division, Department of Medicine, Faculty of Medicine, Khon Kaen University, Khon Kaen, TH; 3 North-Eastern Stroke Research Group, Khon Kaen University, Khon Kaen, TH; 4 Norwich Medical School, University of East Anglia, Norwich, UK; 5 Ambulatory Medicine Division, Department of Medicine, Faculty of Medicine, Khon Kaen University, Khon Kaen, TH; 6 Clinical Informatics, Department of Medicine, University of Cambridge, Cambridge, UK; 7 Keele Cardiovascular Research Group, Centre for Prognosis Research, Institute for Primary Care and Health Sciences, Keele University, Stoke-on-Trent, UK

**Keywords:** diabetes mellitus, stroke, mortality, recurrence, complications, Thailand

## Abstract

**Background:**

Patients with diabetes mellitus (DM) have been found to be at an increased risk of suffering a stroke. However, research on the impact of DM on stroke outcomes is limited.

**Objectives:**

We aimed to examine the influence of DM on outcomes in ischaemic (IS) and haemorrhagic stroke (HS) patients.

**Methods:**

We included 608,890 consecutive stroke patients from the Thailand national insurance registry. In-hospital mortality, sepsis, pneumonia, acute kidney injury (AKI), urinary tract infection (UTI) and cardiovascular events were evaluated using logistic regressions. Long-term analysis was performed on first-stroke patients with a determined pathology (n = 398,663) using Royston-Parmar models. Median follow-ups were 4.21 and 4.78 years for IS and HS, respectively. All analyses were stratified by stroke sub-type.

**Results:**

Mean age (SD) was 64.3 (13.7) years, 44.9% were female with 61% IS, 28% HS and 11% undetermined strokes. DM was associated with in-hospital death, pneumonia, sepsis, AKI and cardiovascular events (odds ratios ranging from 1.13–1.78, p < 0.01) in both stroke types. In IS, DM was associated with long-term mortality and recurrence throughout the follow-up: HR_max_ (99% CI) at t = 4108 days: 1.54 (1.27, 1.86) and HR (99% CI) = 1.27(1.23,1.32), respectively. In HS, HR_max_ (t = 4108 days) for long-term mortality was 2.10 (1.87, 2.37), significant after day 14 post-discharge. HR_max_ (t = 455) for long-term recurrence of HS was 1.29 (1.09, 1.53), significant after day 116 post-discharge.

**Conclusions:**

Regardless of stroke type, DM was associated with in-hospital death and complications, long-term mortality and stroke recurrence.

## Introduction

Diabetes mellitus (DM) is an ever-growing global threat for public health, being a significant source of morbidity and mortality [1]. Patients with DM have been found to have an increased risk of overall stroke incidence and recurrence [2,3,4,5,6,7,8]. Previous studies have also confirmed that patients with diabetes are more likely to experience functional disability and suffer physical limitations after stroke [9]. However, research on the influence of DM on stroke outcomes, stratifying by event type (either ischaemic -IS or haemorrhagic -HS), is currently limited.

Differences in the epidemiology of stroke types between people with and without diabetes have been previously studied. In those with diabetes, ischaemic strokes are more common compared to those without diabetes [10]. Patients with DM have been found to have an increased risk of ischaemic stroke compared to those without DM [10]. Furthermore, the proportion of patients with diabetes is higher in ischaemic stroke patients compared to haemorrhagic stroke patients [11]. This may be due to long-term consequences of DM, such as thrombotic complications and microvascular dysfunction. This suggests there are different pathological processes underlying the outcomes of the two types of stroke in patients with diabetes which may result in different outcomes.

In the current study, we aimed to compare the demographic characteristics and relevant post-stroke outcomes (in-hospital complications, short and long-term mortality and stroke recurrence) between patients with DM and those without DM diagnosed with IS, HS and undetermined strokes in a large, prospectively collected, insurance-based multi-centre stroke register database from Thailand [12].

## Methods

### Data source

We performed this cohort study using the Universal Coverage Health Security Insurance Scheme Database from Thailand, which covers around 75% of the population of Thailand. All patients included were admitted to hospitals across Thailand between September 2004 and September 2015 (follow-up data available until end of December 2015) with a diagnosis of stroke. The primary diagnosis, comorbidities and in-hospital complications were coded in the database using ICD10 – International Classification of Disease, tenth version. The ICD10 codes I61, I63 and I64 were utilised to extract primary diagnoses of intracerebral haemorrhage, cerebral infarction and stroke of undetermined pathology, respectively.

The data source has been previously described [12]. Briefly, in Thailand, the diagnosis of stroke is made during hospitalisation by attending clinical teams based on the clinical features and investigation findings, including brain imaging. Comorbidities (hypertension [I10], heart failure [I50], atrial fibrillation [I48], anaemia [D50; D53; D56; D58; D59], hyperlipidaemia [I67.5], rheumatic valve disease [I09.9], ischaemic heart disease [I25], arrhythmias [I49; R00], chronic kidney disease [N18], liver disease [K76.9], epilepsy [G40], chronic obstructive pulmonary disease [J44]), DM [E10; E11], stroke type [I61; I63; I64], age, sex, length of stay, post-discharge mortality, in-hospital complications (sepsis [A40; A41]; pneumonia [J14, J15, J18]; cardiovascular events – myocardial infarction [I21], cardiac arrest [I46] and recurrent stroke; urinary tract infection [N39] and acute kidney injury [N17.9]) statuses were obtained from reimbursement forms on an annual basis. ICD codes are assigned to each patient covered by the Universal Coverage Health Security Insurance Scheme admitted to a public hospital by the admitting clinician based on pre-existing co-morbidities and acute diagnoses.

In the long-term analysis, only type-specific recurrent strokes were considered. In order to account for new diagnoses of DM after the incident stroke, DM was introduced as a binary time-updated variable. The comorbidity codes of readmissions after the index stroke were analysed and the DM yes/no status was changed accordingly to account for incident diabetes.

This study protocol conforms to the ethical guidelines of the 1975 Declaration of Helsinki as reflected in a priori approval by the Ethics Committee in Human Research, Khon Kaen University, Khon Kaen, Thailand.

### Eligibility criteria

**Figure 1:**
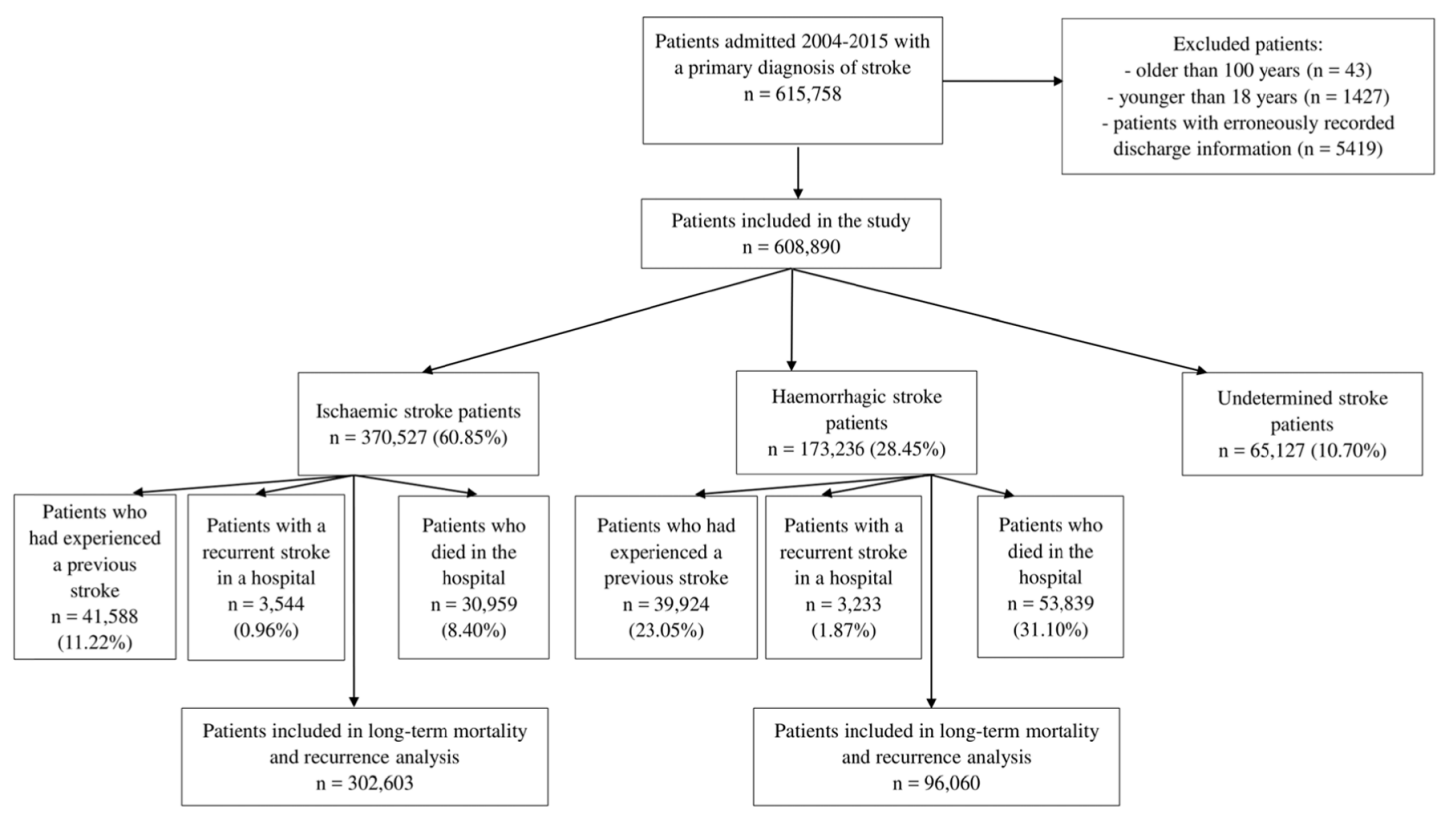
Patient population flowchart. Patients may be classified in one or more exclusion groups and thus the total number of excluded patients does not equal the sum across individual groups.

We excluded patients older than 100 years or younger than 18 years of age and patients with missing post-discharge information. For the long-term analyses, we additionally excluded patients with undetermined strokes, as it is not possible to define type-specific recurrent strokes in these patients. We also excluded patients who died in the hospital and those who already had a recurrent stroke in the hospital, as the first recurrent stroke was considered an end-point of that part of the analysis. For the same reason, patients who had experienced a previous stroke were excluded in the long-term analysis (Figure 1).

### Statistical analysis

Statistical analysis was conducted using SPSS for Windows version 24.0 (SPSS Inc, Chicago, IL) and Stata 14.2 SE (StataCorp 2015), Stata Statistical Software. Given our large sample size, a *P* value < 0.01 was set as the threshold for statistical significance for all analyses. Descriptive statistics for stroke patients were presented for patients without DM and those with DM and stratified by stroke type. Patient characteristics on admission were presented as mean values with standard deviation (SD) for age, medians with interquartile ranges (IQR) for length of stay and as frequencies (%) for categorical variables. Between-group comparisons were performed using the independent samples t-test for age, Mann-Whitney test for the length of stay and the χ^2^ test for categorical variables.

Binomial logistic regressions were performed to examine the impact of DM on the development of in-hospital complications (sepsis, pneumonia, Urinary Tract Infections – UTI, cardiovascular events and Acute Kidney Injury – AKI) and mortality. Multivariable models were sequentially adjusted as follows: model A – unadjusted; model B – adjusted for sex and age; model C – adjusted for sex, age and pre-existing medical conditions; model D – adjusted for sex, age and pre-existing medical conditions and other in-hospital complications (Supplementary Table 5). All regression models were stratified by stroke type.

In the long-term analysis, patients were followed up from the date of discharge from the hospital. Flexible parametric survival regression models were employed to analyse the long-term outcomes (post-discharge mortality and recurrence), as these are more flexible than the standard Cox models in dealing with non-proportional hazards [13]. The proportional hazards assumption for the diabetes variable was verified visually using log-negative-log plots. Where the assumption was not met, the respective variable was introduced in the model as a time-varying co-variate using restricted cubic splines (RCSs). The optimal number of internal knots used to model each variable using RCSs was chosen based on the best-fitting model, as determined visually and using the Akaike’s and Bayesian Information Criteria. We used four internal knots for ischaemic and haemorrhagic stroke mortality and three for haemorrhagic stroke recurrence.

In-hospital analyses were adjusted for age, sex, and pre-existing co-morbidities (atrial fibrillation, chronic kidney injury, chronic obstructive pulmonary disease, heart failure, hypertension, ischaemic heart disease, liver disease, epilepsy, arrhythmias, anaemia, hyperlipidaemia and rheumatic valve disease) and in-hospital complications (acute kidney injury, urinary tract infection, sepsis, pneumonia, cardiovascular events).

The long-term recurrence analyses were adjusted for age, sex and pre-existing-comorbidities (atrial fibrillation, chronic kidney injury, chronic obstructive pulmonary disease, heart failure, hypertension, ischaemic heart disease, liver disease, epilepsy, arrhythmias, anaemia, hyperlipidaemia, rheumatic valve disease). The long-term mortality analyses were adjusted for age, sex, comorbidities and recurrent stroke. In order to reduce the bias due to the selection of covariates, the ones included in the models were chosen based on previous literature [2,14,15,16,17,18,19].

Additional analyses including an interaction term between sex and diabetes status were performed for all short- and long-term outcomes in order to explore any potential sex differences. Further sub-analyses stratifying for subtypes of diabetes mellitus (type 1 and type 2) were undertaken.

## Results

A total of 615,758 patients were admitted with a primary diagnosis of stroke during the study period. After the exclusion of subjects older than 100 years (n = 43), younger than 18 years (n = 1427) and patients with missing post-discharge information (n = 5419), a total of 608,890 patients (99% of total sample) were included in the current study. For the long-term mortality and recurrence analysis we further excluded patients with undetermined stroke subtypes (n = 65,127), those who had experienced a previous stroke event (41,588 for ischaemic, 39,924 for haemorrhagic), individuals who died during their first stroke admission (30,959 for ischaemic, 53,839 for haemorrhagic) and those with recurrent stroke in the hospital (3,544 for ischaemic and 3,233 for haemorrhagic). After applying additional exclusion criteria, the long-term analysis sample consisted of 398,663 (65% of total sample) individuals.

There were 370,527 patients (60.85%) diagnosed with an ischaemic and 173,236 patients (28.45%) with a haemorrhagic stroke. There were only 65,127 (10.7%) patients diagnosed with an undetermined stroke. The mean (SD) age was 64.3 ± 13.7 years and the median (IQR) length of stay was 4 (2,7) days. For the 398,663 individuals remaining in the long-term analysis, median follow-up time was 4.21 years for ischaemic stroke and 4.78 for haemorrhagic stroke. The maximum follow-up was 11.25 years. The prevalence of DM in our cohort was 17.1% (n = 104,128). In the ischaemic stroke group, 75,434 (20.4%) patients had DM. In the haemorrhagic stroke group, there were 16,917 (9.8%) patients with DM. In the undetermined stroke group, there were 11,777 (18.1%) patients with DM, demonstrating similar profile as in ischaemic stroke sub-type.

### Characteristics

Tables 1, 2 and Supplementary Table 1 present the baseline characteristics of patients admitted with a primary diagnosis of ischaemic, haemorrhagic or undetermined stroke. In the ischaemic and undetermined stroke groups, patients without DM were older than those with DM. In the haemorrhagic stroke group patients with DM group were older than those without. All differences in age were statistically significant. There was a significantly greater percentage of female patients with DM amongst all stroke types.

**Table 1:** Characteristics of hospitalized ischaemic stroke patients in Thailand.

Variable	No diabetes mellitus (295,093)	Diabetes mellitus (75,434)	*p*-value

Age, mean ± Standard Deviation	65.8 ± 13.7	64.6 ± 11.3	<0.001
Length of Stay, median (interquartile range)	3 (2–6)	4 (2–7)	<0.001
Post-discharge mortality, N (%)	110,800 (37.5)	30,657 (40.6)	<0.001
Recurrent strokes, N (%)	27,353 (9.3)	8141 (10.8)	<0.001
Female, N (%)	129,152 (43.8)	43,648 (57.9)	<0.001
Male, N (%)	165,941 (56.2)	31,786 (42.1)	
Hypertension, N (%)	121,183 (41.1)	55,061 (73.0)	<0.001
Heart Failure, N (%)	8793 (3.0)	2729 (3.6)	<0.001
Atrial Fibrillation, N (%)	28,436 (9.6)	5034 (6.7)	<0.001
Anaemia, N (%)	17,361 (5.9)	6934 (9.2)	<0.001
Hyperlipidaemia, N (%)	80,909 (27.4)	34,303 (45.5)	<0.001
Rheumatic Valve Disease, N (%)	994 (0.3)	79 (0.1)	<0.001
Ischaemic Heart Disease, N (%)	9606 (3.3)	4451 (5.9)	<0.001
Arrhythmia, N (%)	31,402 (10.6)	5684 (7.5)	<0.001
Chronic kidney disease, N (%)	14,109 (4.8)	8607 (11.4)	<0.001
Liver disease, N (%)	2983 (1.0)	836 (1.1)	0.018
Epilepsy, N (%)	3731 (1.3)	862 (1.1)	0.007
Chronic Obstructive Pulmonary Disease, N (%)	6530 (2.2)	889 (1.2)	<0.001
Pneumonia, N (%)	25,679 (8.7)	7039 (9.3)	<0.001
Sepsis, N (%)	7819 (2.6)	3058 (4.1)	<0.001
Urinary tract infection, N (%)	986 (0.3)	407 (0.5)	<0.001
Cardiovascular events, N (%)	30,861 (10.5)	9380 (12.4)	<0.001
Acute Kidney Injury, N (%)	9754 (3.3)	4241 (5.6)	<0.001
Death, N (%)	24,266 (8.2)	6693 (8.9)	<0.001

**Table 2:** Characteristics of hospitalized haemorrhagic stroke patients in Thailand.

Variable	No diabetes mellitus (156,319)	Diabetes mellitus (16,917)	*p*-value

Age, mean ± Standard Deviation	61 ± 14.5	62.3 ± 11.8	<0.001
Length of Stay, median (interquartile range)	5 (2–11)	6 (2–14)	<0.001
Post-discharge mortality, N (%)	50,232 (32.1)	5893 (34.8)	<0.001
Recurrent strokes, N (%)	8328 (5.3)	936 (5.53)	0.26
Female, N (%)	60,693 (38.8)	9135 (54.0)	<0.001
Male, N (%)	95,626 (61.2)	7782 (46.0)	
Hypertension, N (%)	80,837 (51.7)	14,007 (82.8)	<0.001
Heart Failure, N (%)	1926 (1.2)	538 (3.2)	<0.001
Atrial Fibrillation, N (%)	2882 (1.8)	544 (3.2)	<0.001
Anaemia, N (%)	12,069 (7.7)	2478 (14.6)	<0.001
Hyperlipidaemia, N (%)	11,417 (7.3)	4070 (24.1)	<0.001
Rheumatic Valve Disease, N (%)	132 (0.1)	17 (0.1)	0.499
Ischaemic Heart Disease, N (%)	1851 (1.2)	689 (4.07)	<0.001
Arrhythmia, N (%)	3863 (2.5)	706 (4.2)	<0.001
Chronic kidney disease, N (%)	4976 (3.2)	2229 (13.2)	<0.001
Liver disease, N (%)	2896 (1.9)	315 (1.9)	0.93
Epilepsy, N (%)	1669 (1.1)	207 (1.2)	0.06
Chronic Obstructive Pulmonary Disease, N (%)	2067 (1.3)	166 (1.0)	<0.001
Pneumonia, N (%)	19,685 (12.6)	2865 (16.9)	<0.001
Sepsis, N (%)	5049 (3.2)	1024 (6.1)	<0.001
Urinary tract infection, N (%)	375 (0.2)	76 (0.4)	<0.001
Cardiovascular events, N (%)	11,249 (7.2)	1406 (8.3)	<0.001
Acute Kidney Injury, N (%)	4084 (2.6)	1065 (6.3)	<0.001
Death, N (%)	48,520 (31.0)	5319 (31.44)	0.28

Across all stroke types, patients with DM had a higher post-discharge mortality and longer in-hospital stay than patients without DM. In most cases, patients with DM were more likely to have pre-existing comorbidities.

### In-hospital complications

Table 3 displays the odds ratios and 99% confidence intervals for developing complications during the hospital stay: pneumonia, sepsis, cardiovascular events (myocardial infarction, cardiac arrest and recurrent stroke – only for ischaemic and haemorrhagic stroke patients), urinary tract infection, acute kidney injury and in-hospital death in patients with DM. Patients without diabetes served as a reference category. The results are presented stratified by stroke subtype. Adjustment only for age, sex and comorbidities but not complications did not change the results compared to the fully adjusted models (Supplementary Tables 2, 3 and 4).

**Table 3:** Odds ratios with 99% confidence intervals of developing different stroke complications in patients with diabetes mellitus in ischaemic, haemorrhagic and undetermined stroke patients in Thailand.

Ischaemic Stroke		

	Diabetes mellitus vs. no diabetes mellitus	

Complication	Odds Ratio (99% Confidence Intervals)	*p*-value

Pneumonia	**1.19 (1.15, 1.24)**	**<0.001**
Sepsis	**1.57 (1.47, 1.67)**	**<0.001**
Urinary tract infection	**1.34 (1.13, 1.57)**	**<0.001**
Cardiovascular events	**1.21 (1.16, 1.25)**	**<0.001**
Acute Kidney Injury	**1.53 (1.45, 1.62)**	**<0.001**
In-hospital death	**1.13 (1.08, 1.18)**	**<0.001**
**Haemorrhagic stroke**		

	**Diabetes mellitus vs. no diabetes mellitus**	

**Complication**	**Odds Ratio (99% Confidence Intervals)**	***p*-value**

Pneumonia	**1.15 (1.08, 1.23)**	**<0.001**
Sepsis	**1.55 (1.40, 1.72)**	**<0.001**
Urinary tract infection	1.25 (0.88, 1.76)	0.099
Cardiovascular events	**1.14 (1.05, 1.24)**	**<0.001**
Acute Kidney Injury	**1.78 (1.60, 1.97)**	**<0.001**
In-hospital death	**1.13 (1.08, 1.19)**	**<0.001**
**Undetermined stroke**		

	**Diabetes mellitus vs. no diabetes mellitus**	

**Complication**	**Odds Ratio (99% Confidence Intervals)**	***p*-value**

Pneumonia	**1.24 (1.10, 1.41)**	**<0.001**
Sepsis	**1.43 (1.18, 1.73)**	**<0.001**
Urinary tract infection	1.54 (0.99, 2.42)	0.013
Cardiovascular events	1.16 (0.87, 1.54)	0.186
Acute Kidney Injury	**1.41 (1.18, 1.68)**	**<0.001**
In-hospital death	0.93 (0.81, 1.06)	0.13

In patients with ischaemic strokes, DM was associated with increased odds of developing all presented complications (all data presented as OR (99% CI) for the fully adjusted models): pneumonia 1.19 (1.15, 1.24), sepsis 1.57 (1.47, 1.67), urinary tract infection 1.34 (1.13, 1.57), cardiovascular events 1.21 (1.16, 1.25) and acute kidney injury 1.53 (1.45, 1.62). In the haemorrhagic stroke cohort, there was an association between DM and higher odds of developing pneumonia 1.15 (1.08, 1.23), sepsis 1.55 (1.40, 1.72), cardiovascular events 1.14 (1.05, 1.24) and acute kidney injury 1.78 (1.60, 1.97). There was no significant association between DM and incident urinary tract infection in the fully adjusted model. Patients in the undetermined stroke group with DM had increased odds of pneumonia 1.24 (1.10, 1.41), sepsis 1.43 (1.18, 1.73) and acute kidney injury 1.41 (1.18, 1.68), compared to patients without DM. There was no association between DM and urinary tract infection or cardiovascular events amongst undetermined stroke patients.

### In-hospital mortality

Whilst DM was a significant risk factor for in-hospital death in ischaemic and haemorrhagic strokes (1.13 [1.08, 1.18] and 1.13 [1.08, 1.19], respectively), it was not a significant outcome for patients with an undetermined stroke (Table 3).

### Long-term mortality and recurrence

**Figure 2:**
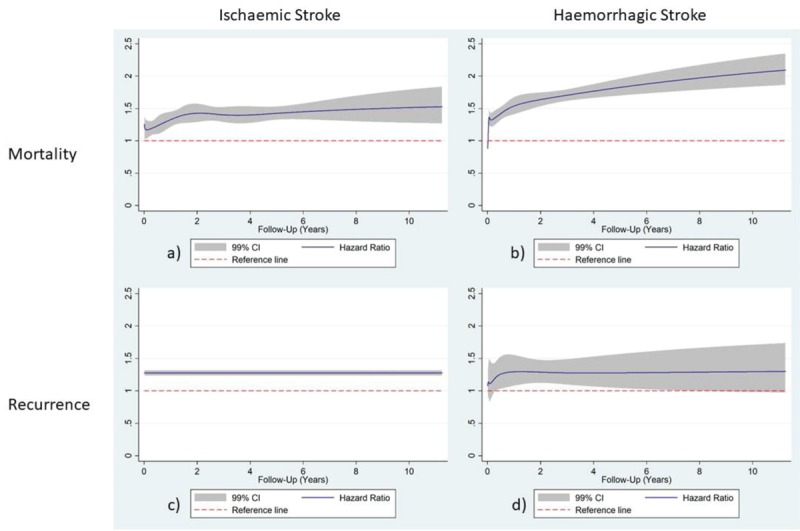
Hazard Ratio functions have been plotted against post-discharge follow-up time (days) using RCS modelling with 99% confidence intervals for mortality in ischaemic stroke diabetes mellitus patients **(a)**, mortality in haemorrhagic stroke diabetes mellitus patients **(b)**, recurrence in ischaemic stroke patients **(c)** and recurrence in haemorrhagic stroke diabetes mellitus patients **(d)** in Thailand; patients without diabetes were used as a reference category.

The results of the survival analyses are presented in Table 4 and Figure 2. In patients diagnosed with ischaemic and haemorrhagic stroke, DM was associated with higher long-term mortality and recurrent events.

**Table 4:** Death and recurrent stroke events and Hazard Ratios with 99% confidence intervals in ischaemic and haemorrhagic stroke patients with diabetes mellitus.

Ischaemic stroke			

	Diabetes mellitus		No diabetes mellitus

	Events (%)	Hazard Ratio (99% Confidence Intervals)	Events (%)

Death	29,135 (44.3%)	†	99,715 (42.1%)
Recurrent stroke	7679 (11.7%)	1.52 (1.26, 1.84)	23,539 (9.9%)
**Haemorrhagic stroke**			

	**Diabetes mellitus**		**No diabetes mellitus**

	**Events (%)**	**Hazard Ratio (99% Confidence Intervals)**	**Events (%)**

Death	4841 (50.1%)	†	40,495 (46.9%)
Recurrent stroke	758 (7.8%)	†	6546 (7.6%)

† Proportional hazards assumption not met (see Figure 2).

Due to non-compliance with the proportional hazards assumption, some of the exposure variables in some of the analysed subgroups were modelled using RCSs, resulting in non-constant Hazard Ratios over the follow-up time, which are presented as such. In the ischaemic stroke group (Figure 2a), mortality was significantly higher in patients with DM throughout the follow-up time, increasing constantly until reaching a maximum at the end of the follow-up period: maximum Hazard Ratio (HR_max_) (99% CI) (t = 4108) 1.54 (1.27, 1.86). For ischaemic stroke recurrence, DM was associated with the outcome throughout the follow-up time (1.27 (1.23, 1.32)). In the haemorrhagic group, mortality was significantly higher in DM patients after the 14^th^ day post-discharge hazard ratio (HR) (t = 14 days) 1.23 (1.09, 1.40), with the maximum HR (t = 4108 days) 2.10 (1.87, 2.37). Furthermore, DM was associated with haemorrhagic stroke recurrence after the 116^th^ day (HR (t = 116 days) 1.21 (1.00, 1.46)), reaching the maximum on the 455th day HR (t = 455 days) 1.29 (1.09, 1.53). The function becomes non-significant on the 2648th day of follow-up (HR (t = 2648 days) 1.28 (1.00, 1.65)).

### Analysis of sex differences

The results of the analyses including the interaction term between sex (female vs. male) and diabetes status are presented in the Supplementary Tables 6 and 7. For the in-hospital outcomes, women had a lower risk of pneumonia OR (99% CI): 0.91 (0.84–0.98) and cardiovascular events 0.83 (0.70–0.99), but a higher risk of AKI 1.16 (1.04–1.28) compared to males. In long-term outcomes analysis, women had a lower risk of ischaemic stroke mortality, HR (99% CI): 0.95 (0.92–0.98).

### Sub-group analysis

The results of the sub-group analyses stratified by type of diabetes (type 1 and type 2) are presented in Supplementary Tables 8 and 9 as well as in Supplementary Figure 1. In this cohort, T1DM was not associated with any of the short-term outcomes or long-term recurrence, in all stroke types. However, T1DM was associated with long-term mortality in both ischaemic HR (99% CI): 1.89 (1.54–2.31) and haemorrhagic stroke 1.84 (1.21–2.80). The results for the T2DM subgroup analyses were similar to the results of the main analyses.

## Discussion

For both ischaemic and haemorrhagic strokes, DM was associated with higher in-hospital and long-term mortality and recurrence. Furthermore, our results highlight the positive association between DM and the development of certain in-hospital complications (pneumonia, sepsis, urinary tract infection, cardiovascular events – myocardial infarction, cardiac arrest, recurrent stroke event, and acute kidney injury) for both stroke subtypes. For both stroke subtypes, DM was associated with recurrence, which can be explained by the fact that patients with DM are more likely to be overweight and have a higher cardiovascular disease burden [20].

Our additional analyses which included the sex*diabetes interaction term confirmed a lower risk of some of the outcomes (pneumonia, cardiovascular events, long-term mortality) for women compared to men with diabetes mellitus. Nevertheless, women with diabetes had a higher risk of AKI than men. Previous studies have yielded equivocal results regarding the interaction between sex and diabetes mellitus in stroke outcomes, which could be due to ethnic differences between populations [21,22,23]. More research is needed to clearly establish the relationship between diabetes, sex and stroke outcomes in different populations. The analysis stratified by diabetes mellitus type (type 1 and type 2) showed that whilst the results for patients with type 2 DM were similar to the overall results, significant associations were observed only for long-term mortality in patients with type 1 DM. Given the relatively small number of patients with type 1 DM in this cohort, it is likely that the lack of association between type 1 diabetes mellitus and short-term outcomes and long-term recurrence may be due to type II error.

Our finding that DM is positively associated with increased risk of long-term mortality and recurrence was consistent with previous studies [8,18,24,25,26,27,28]. One of them reported that the survival of a patient with DM after a stroke in years 2000–2005 was very similar to the survival of a patient without DM after a stroke between 1985 and 1989 [25]. Our results highlight this disparity after both ischaemic and haemorrhagic stroke.

Nevertheless, in contrast to some of the previous studies, we found that in-hospital mortality is increased in patients with DM in both ischaemic and haemorrhagic stroke [29,30]. Given that the previous studies were performed on much smaller samples, it may be the case that their findings may be due to a type II error. Furthermore, treatment differences between Europe and Thailand, as well as differences in other population level factors such as general health, ethnicity, and other social determinants etc. may also explain those differences.

Asia is a continent of high ethnic variation and cultural diversity. Apart from generally higher cardiovascular burden compared to Western populations, Asian populations have a higher incidence of stroke as well as higher stroke mortality [31]. There is currently a lack of studies assessing the incidence of stroke in Thailand specifically. However, the prevalence of stroke in Thailand is around 2.0%, which was lower than in countries like Singapore, Korea and India [32]. It has been established that the incidence of haemorrhagic stroke is higher amongst Asian populations compared to their Western counterparts [33]. Nevertheless, the Thai population have been found to have the highest proportion of ischaemic strokes amongst East, South and South-East Asian countries [34]. Given such a high heterogeneity of stroke epidemiology in Asia, it is important to consider these differences when interpreting our results in the context of other stroke populations.

Understanding the mechanisms behind the increased complication rates, mortality and stroke recurrence in stroke patients with DM is required in order to devise strategies aimed at improving the outcomes of this substantial patient population. Previous translational studies suggest a few possible mechanisms which may potentially account for our findings. Vascular damage caused by the endogenous cardiovascular nitric oxide system and increased accumulation of lactate caused by anaerobic glucose metabolism are some mechanisms which occur more readily in patients with DM and may be linked to the adverse mortality outcomes [8]. Furthermore, the increased risk of recurrent strokes and death in patients with diabetes may be the result of vascular changes caused by the synergistic effect of chronic hyperglycaemia, free fatty acids release and insulin resistance [35]. The imbalance between vasodilator and vasoconstrictors in the peripheral circulation may also be a factor that is responsible for the adverse cardiovascular outcomes in stroke patients with diabetes. It has been shown that hyperglycaemia inhibits activation of endothelial nitric oxide synthase, resulting in either a reduction of nitric oxide production or a decrease in its bioavailability. Low nitric oxide levels limit vessel dilatation and relaxation as well as favour platelet adhesion to the vascular intimal surface, thus intensifying the progression of atherosclerosis and predisposing to plaque rupture. Furthermore, DM is associated with an increased production of vasoconstrictors, such as endothelin-1. These may not only induce vasoconstriction, but also stimulate the renin-angiotensin system and cause vascular smooth muscle hypertrophy [35].

It is perhaps not surprising that patients with diabetes are at higher risk of developing acute kidney injury, given that they are more likely to have an impaired renal function [36,37]. Increased susceptibility to infections such as urinary tract infections, pneumonia and sepsis in patients with diabetes can be caused by the effects of hyperglycaemia on the immune system, such as disrupted chemotaxis, impaired phagocytosis, reduced break-down of phagocytosed organisms and altered adherence of micro-organisms to polymorphonuclear leukocytes and lymphocytes [38]. Furthermore, in the particular case of urinary tract infections, sequelae of DM such as glycosuria and autonomic neuropathy-associated urinary retention increase the susceptibility of patients with diabetes [39,40].

This is the first study to perform a comprehensive analysis of the characteristics, complications, mortality and recurrence in patients with ischaemic and haemorrhagic strokes, using robust statistical methodology. Whilst post-stroke outcomes have been examined in several previous studies, these studies are subject to several limitations. They did not study outcomes by stroke type, included low number of subjects or had a shorter follow-up period for long term analysis, had more limiting age ranges as an exclusion criterion than our study, and did not account for any incident diagnoses of DM [2,8,18,25,26,27,28,29,41]. The present study provides a detailed description of the effect of diabetes on outcomes after ischaemic and haemorrhagic stroke in a large unselected sample, with a long follow-up period in long-term mortality and recurrence analysis. We also accounted for incident DM diagnoses during post-discharge follow-up time, which improved the accuracy of models in our study.

We acknowledge certain study limitations. Firstly, due to unavailable data, we could not adjust for other potential confounding factors such as: diet, BMI, smoking, excessive alcohol drinking, sedentary lifestyle, low income, urbanization or use of preventative medications [42,43]. Similarly, we were unable to examine the effect of stroke severity, glycaemic control and therapy. We were also unable to adjust for severity of AKI, as it is not quantified by ICD-10 codes. Due to differences in treatment, social factors, population health and ethnicity between Thailand and Western countries, the results of our study may not be applicable to non-Asian populations. The increased long-term mortality of DM patients confirmed by the results of our study shows that despite advances in stroke care in Thailand, there is still a disparity between patients with and without diabetes. Our results highlight the importance of further research on adapting post-stroke care to the needs of patients with diabetes in order to prevent stroke recurrence and reduce long-term mortality.

## Conclusion

In the largest cohort study on stroke outcomes in patients with diabetes to date, we found that DM is positively associated with higher risk of post-stroke complications in all stroke patients as well as in-hospital death in ischaemic and haemorrhagic stroke patients. Long-term mortality and recurrence after stroke were increased amongst patients with diabetes in both stroke subtypes. More research is needed to tailor preventative and curative therapies to improve the clinical outcome for the stroke patients with DM, as stroke remains one of the leading causes of mortality and morbidity as well as an important cause of disability worldwide.

## Additional File

The additional file for this article can be found as follows:

10.5334/gh.364.s1 Supplementary material. Supplementary tables and figures.
