# Supplementary material for: Impact of Diabetes on Complications, Long Term Mortality and Recurrence in 608,890 Hospitalised Patients with Stroke
Source: Glob Heart. 2020 Feb 6;15(1):2. doi: 10.5334/gh.364 (PMC7218766; doi:10.5334/gh.364)
Supplement: Supplementary material. — Supplementary tables and figures. [file gh-15-1-364-s1.pdf]

Variable	No diabetes mellitus (53,350)	Diabetes mellitus (11,777)	*p*-value
Age, mean ± Standard Deviation	65.7±13.9	64.7±11.0	<0.001
Length of Stay, median (interquartile range)	3(2-5)	3(2-5)	0.002
Post-discharge mortality, N (%)	25633 (48.0)	6099 (51.8)	<0.001
Female, N (%)	23,892 (44.8)	4724 (40.1)	<0.001
Male, N (%)	29,458 (55.2)	7053 (59.9)	
Hypertension, N (%)	18833 (35.3)	8016 (68.1)	<0.001
Heart Failure, N (%)	1113 (2.1)	298 (2.5)	0.003
Atrial Fibrillation, N (%)	2954 (5.5)	398 (3.4)	<0.001
Anaemia, N (%)	2302 (4.3)	855 (7.3)	<0.001
Hyperlipidaemia, N (%)	8912 (16.7)	3952 (33.6)	<0.001
Rheumatic Valve Disease, N (%)	111 (0.2)	12 (0.1)	0.02
Ischaemic Heart Disease, N (%)	1422 (2.7)	542 (4.6)	<0.001
Arrhythmia, N (%)	3373 (6.3)	460 (3.9)	<0.001
Chronic kidney disease, N (%)	2053 (3.8)	1284 (10.9)	<0.001
Liver disease, N (%)	447 (0.8)	132 (1.1)	0.003
Epilepsy, N (%)	690 (1.3)	123 (1.0)	0.03
Chronic Obstructive Pulmonary Disease, N (%)	1105 (2.1)	130 (1.1)	<0.001
Pneumonia, N (%)	2865 (5.4)	673 (5.7)	0.14
Sepsis, N (%)	963 (1.8)	309 (2.6)	<0.001
Urinary tract infection, N (%)	133 (0.2)	54 (0.5)	<0.001
Cardiovascular events, N (%)	433 (0.8)	126 (1.1)	0.01
Acute Kidney Injury, N (%)	1001 (1.9)	362 (3.1)	<0.001
Death, N (%)	3015 (5.7)	598 (5.1)	0.01

Supplementary Table 1 Baseline characteristics of hospitalized undetermined stroke patients in Thailand

Complication	Diabetes mellitus	
Pneumonia	Odds Ratio (99% Confidence Intervals)	*p*-value
A	**1.08 (1.04, 1.12)**	**<0.001**
B	**1.18 (1.13, 1.22)**	**<0.001**
C	**1.26 (1.21, 1.31)**	**<0.001**
D	**1.19 (1.15, 1.24)**	**<0.001**
Sepsis	Odds Ratio (99% Confidence Intervals)	*p*-value
A	**1.55 (1.47, 1.64)**	**<0.001**
B	**1.57 (1.48, 1.66)**	**<0.001**
C	**1.63 (1.53, 1.73)**	**<0.001**
D	**1.57 (1.47, 1.67)**	**<0.001**
Urinary tract infection	Odds Ratio (99% Confidence Intervals)	*p*-value
A	**1.62 (1.39, 1.88)**	**<0.001**
B	**1.51 (1.30, 1.77)**	**<0.001**
C	**1.35 (1.15, 1.59)**	**<0.001**
D	**1.34 (1.13, 1.57)**	**<0.001**
Cardiovascular events	Odds Ratio (99% Confidence Intervals)	*p*-value
A	**1.22 (1.18, 1.26)**	**<0.001**
B	**1.21 (1.17, 1.25)**	**<0.001**
C	**1.20 (1.16, 1.24)**	**<0.001**
D	**1.21 (1.16, 1.25)**	**<0.001**
Acute Kidney Injury	Odds Ratio (99% Confidence Intervals)	*p*-value
A	**1.74 (1.66, 1.83)**	**<0.001**
B	**1.86 (1.77, 1.95)**	**<0.001**
C	**1.63 (1.54, 1.72)**	**<0.001**
D	**1.53 (1.45, 1.62)**	**<0.001**
In-hospital death	Odds Ratio (99% Confidence Intervals)	*p*-value
A	**1.09 (1.05, 1.13)**	**<0.001**
B	**1.09 (1.05, 1.13)**	**<0.001**
C	**1.23 (1.18, 1.28)**	**<0.001**
D	**1.13 (1.08, 1.18)**	**<0.001**

Supplementary Table 2 Odds ratios with 99% CI of developing different stroke complications (pneumonia, sepsis, urinary tract infection, cardiovascular events – cardiac arrest, myocardial infarction and recurrent stroke, acute kidney injury, in-hospital death) in ischaemic stroke patients with diabetes mellitus in Thailand; patients without diabetes mellitus were used as a reference category

Model A – unadjusted

Model B – adjusted for age and sex

Model C – adjusted for age, sex and pre-existing medical conditions (hypertension, heart failure, atrial fibrillation, anaemia, hyperlipidaemia, rheumatic valve disease, ischaemic heart disease, arrhythmia, chronic kidney disease, liver disease, epilepsy, chronic obstructive pulmonary disease)

Model D – adjusted for age, sex, pre-existing medical conditions and other complications

Complication	Diabetes mellitus	
Pneumonia	Odds Ratio (99% Confidence Intervals)	*p*-value
A	**1.42 (1.34, 1.50)**	**<0.001**
B	**1.43 (1.35, 1.51)**	**<0.001**
C	**1.21 (1.14, 1.29)**	**<0.001**
D	**1.15 (1.08, 1.23)**	**<0.001**
Sepsis	Odds Ratio (99% Confidence Intervals)	*p*-value
A	**1.93 (1.76, 2.11)**	**<0.001**
B	**1.90 (1.74, 2.08)**	**<0.001**
C	**1.60 (1.45, 1.77)**	**<0.001**
D	**1.55 (1.40, 1.72)**	**<0.001**
Urinary tract infection	Odds Ratio (99% Confidence Intervals)	*p*-value
A	**1.88 (1.36, 2.60)**	**<0.001**
B	**1.70 (1.23, 2.36)**	**<0.001**
C	1.28 (0.91, 1.81)	0.062
D	1.25 (0.88, 1.76)	0.099
Cardiovascular events	Odds Ratio (99% Confidence Intervals)	*p*-value
A	**1.17 (1.08, 1.26)**	**<0.001**
B	**1.20 (1.11, 1.29)**	**<0.001**
C	**1.14 (1.05, 1.24)**	**<0.001**
D	**1.14 (1.05, 1.24)**	**<0.001**
Acute Kidney Injury	Odds Ratio (99% Confidence Intervals)	*p*-value
A	**2.50 (2.29, 2.74)**	**<0.001**
B	**2.55 (2.32, 2.79)**	**<0.001**
C	**1.86 (1.68, 2.05)**	**<0.001**
D	**1.78 (1.60, 1.97)**	**<0.001**
In-hospital death	Odds Ratio (99% Confidence Intervals)	*p*-value
A	1.02 (0.97, 1.07)	0.28
B	1.01 (0.97, 1.06)	0.55
C	**1.15 (1.10, 1.20)**	**<0.001**
D	**1.13 (1.08, 1.19)**	**<0.001**

Supplementary Table 3 Odds ratios with 99% CI of developing different stroke complications (pneumonia, sepsis, urinary tract infection, cardiovascular events – cardiac arrest, myocardial infarction and recurrent stroke, acute kidney injury, in-hospital death in haemorrhagic stroke patients with diabetes mellitus in Thailand; patients without diabetes mellitus were used as a reference category

Model A – unadjusted

Model B – adjusted for age and sex

Model C – adjusted for age, sex and pre-existing medical conditions (hypertension, heart failure, atrial fibrillation, anaemia, hyperlipidaemia, rheumatic valve disease, ischaemic heart disease, arrhythmia, chronic kidney disease, liver disease, epilepsy, chronic obstructive pulmonary disease)

Model D – adjusted for age, sex, pre-existing medical conditions and other complications

Complication	Diabetes mellitus	
Pneumonia	Odds Ratio (99% Confidence Intervals)	*p*-value
A	1.07 (0.95, 1.20)	0.14
B	**1.18 (1.05, 1.33)**	**<0.001**
C	**1.29 (1.14, 1.45)**	**<0.001**
D	**1.24 (1.10, 1.41)**	**<0.001**
Sepsis	Odds Ratio (99% Confidence Intervals)	*p*-value
A	**1.47 (1.24, 1.74)**	**<0.001**
B	**1.50 (1.26, 1.78)**	**<0.001**
C	**1.50 (1.25, 1.81)**	**<0.001**
D	**1.43 (1.18, 1.73)**	**<0.001**
Urinary tract infection	Odds Ratio (99% Confidence Intervals)	*p*-value
A	**1.84 (1.22, 2.80)**	**<0.001**
B	**1.83 (1.20, 2.78)**	**<0.001**
C	**1.58 (1.01, 2.47)**	**0.009**
D	1.54 (0.99, 2.42)	0.013
Cardiovascular events	Odds Ratio (99% Confidence Intervals)	*p*-value
A	**1.32 (1.02, 1.72)**	**0.006**
B	**1.31 (1.00, 1.70)**	**0.009**
C	1.19 (0.89, 1.58)	0.12
D	1.16 (0.87, 1.54)	0.186
Acute Kidney Injury	Odds Ratio (99% Confidence Intervals)	*p* -value
A	**1.66 (1.41, 1.95)**	**<0.001**
B	**1.75 (1.49, 2.06)**	**<0.001**
C	**1.45 (1.22, 1.73)**	**<0.001**
D	**1.41 (1.18, 1.68)**	**<0.001**
In-hospital death	Odds Ratio (99% Confidence Intervals)	*p* -value
A	0.89 (0.79, 1.00)	0.014
B	0.91 (0.81, 1.02)	0.04
C	0.99 (0.87, 1.13)	0.867
D	0.93 (0.81, 1.06)	0.13

Supplementary Table 4 Odds ratios with 99% CI of developing different stroke complications (pneumonia, sepsis, urinary tract infection, cardiovascular events – cardiac arrest, myocardial infarction and recurrent stroke, acute kidney injury, in-hospital death) in undetermined stroke patients with diabetes mellitus in Thailand; patients without diabetes mellitus were used as a reference category

Model A – unadjusted

Model B – adjusted for age and sex

Model C – adjusted for age, sex and pre-existing medical conditions (hypertension, heart failure, atrial fibrillation, anaemia, hyperlipidaemia, rheumatic valve disease, ischaemic heart disease, arrhythmia, chronic kidney disease, liver disease, epilepsy, chronic obstructive pulmonary disease)

Model D – adjusted for age, sex, pre-existing medical conditions and other complications

Pneumonia				
Variable	Model A	Model B	Model C	Model D
Sex		*	*	*
Age		*	*	*
Hypertension			*	*
Heart failure			*	*
Atrial fibrillation			*	*
Anaemia			*	*
Hyperlipidaemia			*	*
Rheumatic valve disease			*	*
Ischaemic heart disease			*	*
Arrhythmia			*	*
Chronic kidney disease			*	*
Liver disease			*	*
Epilepsy			*	*
Chronic obstructive pulmonary disease			*	*
Pneumonia				
Sepsis				*
Urinary tract infection				*
Cardiovascular events (myocardial infarction, cardiac arrest, recurrent stroke†)				*
Acute kidney injury				*
Sepsis				
Variable	Model A	Model B	Model C	Model D
Sex		*	*	*
Age		*	*	*
Hypertension			*	*
Heart failure			*	*
Atrial fibrillation			*	*
Anaemia			*	*
Hyperlipidaemia			*	*
Rheumatic valve disease			*	*
Ischaemic heart disease			*	*
Arrhythmia			*	*
Chronic kidney disease			*	*
Liver disease			*	*
Epilepsy			*	*
Chronic obstructive pulmonary disease			*	*
Pneumonia				*
Sepsis				
Urinary tract infection				*
Cardiovascular events (myocardial infarction, cardiac arrest, recurrent stroke†)				*
Acute kidney injury				*
Urinary Tract Infection				
Variable	Model A	Model B	Model C	Model D
Sex		*	*	*
Age		*	*	*
Hypertension			*	*
Heart failure			*	*
Atrial fibrillation			*	*
Anaemia			*	*
Hyperlipidaemia			*	*

Rheumatic valve disease			*	*
Ischaemic heart disease			*	*
Arrhythmia			*	*
Chronic kidney disease			*	*
Liver disease			*	*
Epilepsy			*	*
Chronic obstructive pulmonary disease			*	*
Pneumonia				*
Sepsis				*
Urinary tract infection				
Cardiovascular events (myocardial infarction, cardiac arrest, recurrent stroke†)				*
Acute kidney injury				*
Cardiovascular events				
Variable	Model A	Model B	Model C	Model D
Sex		*	*	*
Age		*	*	*
Hypertension			*	*
Heart failure			*	*
Atrial fibrillation			*	*
Anaemia			*	*
Hyperlipidaemia			*	*
Rheumatic valve disease			*	*
Ischaemic heart disease			*	*
Arrhythmia			*	*
Chronic kidney disease			*	*
Liver disease			*	*
Epilepsy			*	*
Chronic obstructive pulmonary disease			*	*
Pneumonia				*
Sepsis				*
Urinary tract infection				*
Cardiovascular events (myocardial infarction, cardiac arrest, recurrent stroke†)				
Acute kidney injury				*
Acute Kidney Injury				
Variable	Model A	Model B	Model C	Model D
Sex		*	*	*
Age		*	*	*
Hypertension			*	*
Heart failure			*	*
Atrial fibrillation			*	*
Anaemia			*	*
Hyperlipidaemia			*	*
Rheumatic valve disease			*	*
Ischaemic heart disease			*	*
Arrhythmia			*	*
Chronic kidney disease			*	*
Liver disease			*	*
Epilepsy			*	*
Chronic obstructive pulmonary disease			*	*
Pneumonia				*
Sepsis				*

Urinary tract infection				*
Cardiovascular events (myocardial infarction, cardiac arrest, recurrent stroke†)				*
Acute kidney injury				
In-hospital mortality				
Variable	Model A	Model B	Model C	Model D
Sex		*	*	*
Age		*	*	*
Hypertension			*	*
Heart failure			*	*
Atrial fibrillation			*	*
Anaemia			*	*
Hyperlipidaemia			*	*
Rheumatic valve disease			*	*
Ischaemic heart disease			*	*
Arrhythmia			*	*
Chronic kidney disease			*	*
Liver disease			*	*
Epilepsy			*	*
Chronic obstructive pulmonary disease			*	*
Pneumonia				*
Sepsis				*
Urinary tract infection				*
Cardiovascular events (myocardial infarction, cardiac arrest, recurrent stroke†)				*
Acute kidney injury				*

Supplementary Table 5 Adjusting covariates included in the complications regression models
†only type-specific recurrent strokes in ischaemic and haemorrhagic groups

Outcome	OR (99%CI)	*p*-value
In-hospital death	0.97 (0.89-1.06)	0.406
UTI	1.21 (0.84-1.72)	0.174
Sepsis	1.03 (0.91-1.16)	0.525
Pneumonia	0.91 (0.84-0.98)	**0.002**
Cardiovascular Events	0.83 (0.70-0.99)	**0.006**
AKI	1.16 (1.04-1.28)	**<0.0001**

Supplementary Table 6 Odds ratios, 99% confidence intervals and p-values for the interaction term for sex (female vs male) and diabetes mellitus in in-hospital outcomes.

Outcome	HR (99%CI)	*p*-value
Ischaemic Stroke Mortality	0.95 (0.92-0.98)	**<0.0001**
Ischaemic Stroke Recurrence	0.96 (0.90-1.03)	0.166
Haemorrhagic Stroke Mortality	0.97 (0.90-1.05)	0.312
Haemorrhagic Stroke Recurrence	1.01 (0.83-1.22)	0.929

Supplementary Table 7 Hazard Ratios, 99% confidence intervals and p-values for the interaction term for sex (female vs male) and diabetes mellitus in long-term stroke outcomes.

Ischaemic stroke				
	Type 1 DM		Type 2 DM	
Complications	OR (99% CI)	*p*-value	OR (99% CI)	*p*-value
Pneumonia	1.70 (0.95, 3.06)	0.02	**1.16 (1.11, 1.20)**	**<0.0001**
Sepsis	2.01 (0.90, 4.48)	0.026	**1.50 (1.41, 1.60)**	**<0.0001**
Urinary Tract Infection	-	-	**1.33 (1.13, 1.56)**	**<0.0001**
Cardiovascular events	1.21 (0.72, 2.04)	0.35	**1.21 (1.17, 1.25)**	**<0.0001**
Acute Kidney Injury	1.05 (0.42, 2.61)	0.9	**1.54 (1.45, 1.62)**	**<0.0001**
In-hospital death	0.729 (0.49, 1.08)	0.038	**1.10 (1.06, 1.15)**	**<0.001**
Haemorrhagic stroke				
	Type 1 DM		Type 2 DM	
Complications	OR (99% CI)	*p*-value	OR (99% CI)	*p*-value
Pneumonia	0.84 (0.36, 1.99)	0.61	**1.11 (1.05, 1.19)**	**<0.0001**
Sepsis	1.98 (0.65, 6.03)	0.11	**1.47 (1.33, 1.63)**	**<0.0001**
Urinary Tract Infection	-	-	1.23 (0.87, 1.74)	0.12
Cardiovascular events	1.60 (0.69, 3.69)	0.15	**1.14 (1.05, 1.23)**	**<0.0001**
Acute Kidney Injury	2.20 (0.75, 6.45)	0.06	**1.78 (1.61, 1.97)**	**<0.0001**
In-hospital death	1.15 (0.62, 1.11)	0.563	**1.12 (1.07, 1.18)**	**<0.001**
Undetermined stroke				
	Type 1 DM		Type 2 DM	
Complication	OR (99% CI)	*p*-value	OR (99% CI)	*p*-value
Pneumonia	0.91 (0.26, 3.18)	0.84	**1.23 (1.08, 1.40)**	**<0.0001**
Sepsis	1.74 (0.40, 7.56)	0.33	**1.40 (1.15, 1.69)**	**<0.0001**
Urinary Tract Infection	-	-	1.56 (0.99, 2.44)	0.011
Cardiovascular events	0.95 (0.07, 13.02)	0.96	1.14 (0.86, 1.52)	0.23
Acute Kidney Injury	1.63 (0.41, 6.53)	0.36	**1.42 (1.19, 1.69)**	**<0.0001**
In-hospital death	1.3 (0.46, 3.64)	0.52	0.91 (0.8, 1.04)	0.08

OR – odds ratio, CI – confidence intervals

Supplementary Table 8 Odds ratios with 99% CI of in-hospital death in patients with type 1 diabetes mellitus and type 2 diabetes mellitus in ischaemic, haemorrhagic and undetermined stroke patients in Thailand; patients without diabetes mellitus were used as a reference category. All the models were adjusted for age, sex, pre-existing medical conditions and complications (sepsis, pneumonia, UTI, cardiovascular events and acute kidney injury).

Ischaemic stroke		
	Type 1 DM	Type 2 DM
Death	**1.89 [1.54-2.31]**	*
Recurrent stroke	1.06 [0.62-1.79]	**1.28 [1.24-1.31]**
Haemorrhagic stroke		
	Type 1 DM	Type 2 DM
Death	**1.84 [1.21-2.80]**	*
Recurrent stroke	2.12 [0.80-4.62]	*

Supplementary Table 9 Death hazard ratios with 99% confidence intervals for stroke patients with type 1, type 2 and without diabetes mellitus in Thailand; patients without diabetes mellitus were used as a reference category;

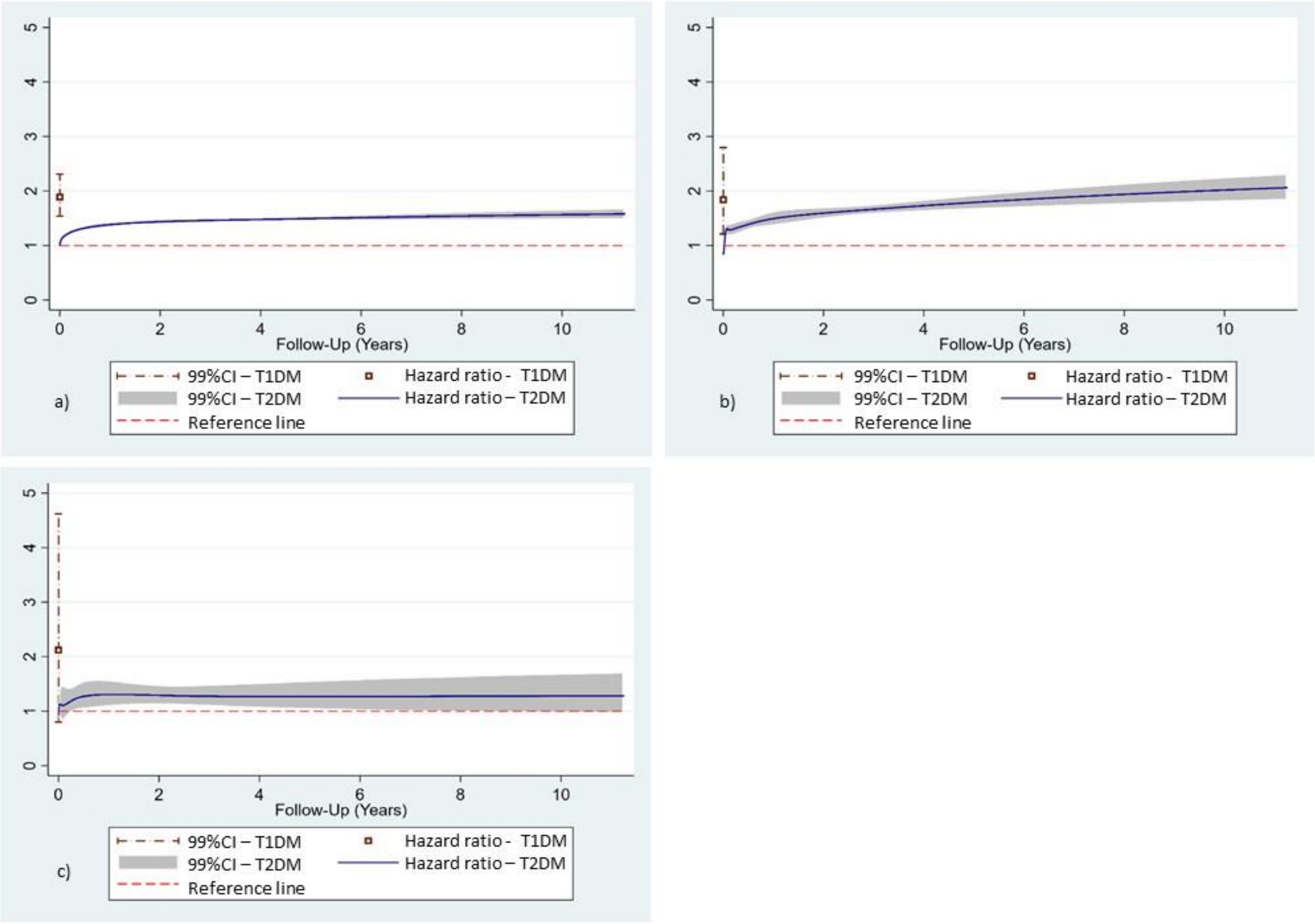

Supplementary Figure 1 Hazard ratio functions plotted against post-discharge follow-up time (days) using RCS modelling with 99% confidence intervals for mortality in ischaemic stroke diabetes mellitus patients (a), mortality in haemorrhagic stroke diabetes mellitus patients (b) and recurrence in haemorrhagic stroke diabetes mellitus patients (c) in Thailand; results in the type 1 patients are represented by the dark red line, results in the type 2 patients are represented by the blue line; the patients without diabetes were used as a reference category. T1DM – type 1 diabetes mellitus, T2CM – type 2 diabetes mellitus, CI – confidence interval
